# Evaluation of the resistome and gut microbiome composition of hospitalized patients in a health unit of southern Brazil coming from a high animal husbandry production region

**DOI:** 10.3389/frabi.2024.1489356

**Published:** 2025-01-17

**Authors:** Elisa Pires Coltro, Lucas Cafferati Beltrame, Caroline Ribeiro da Cunha, Caetana Paes Zamparette, Clarissa Feltrin, Vilmar Benetti Filho, Patrícia de Almeida Vanny, Sérgio Beduschi Filho, Taíse Costa Ribeiro Klein, Mara Cristina Scheffer, Jussara Kasuko Palmeiro, Glauber Wagner, Thaís Cristine Marques Sincero, Carlos Rodrigo Zárate-Bladés

**Affiliations:** ^1^ Center for Dysbiosis Control (CCDis), Federal University of Santa Catarina, Florianopolis, Brazil; ^2^ Laboratory of Immunoregulation (iREG), Department of Microbiology, Immunology and Parasitology, Federal University of Santa Catarina, Florianopolis, Brazil; ^3^ Laboratory of Applied Molecular Microbiology (MIMA), Department of Clinical Analysis, Federal University of Santa Catarina, Florianopolis, Brazil; ^4^ Laboratory of Bioinformatics, Department of Microbiology, Immunology and Parasitology, Federal University of Santa Catarina, Florianopolis, Brazil; ^5^ Intrahospitalar Infection Service, University Hospital Polydoro Ernani de São Thiago, Federal University of Santa Catarina, Florianopolis, Brazil; ^6^ Molecular Biology, Microbiology and Serology, University Hospital Polydoro Ernani de São Thiago, Federal University of Santa Catarina, Florianopolis, Brazil

**Keywords:** antimicrobial resistance genes, resistome, gut microbiome, hospital environment, One Health, metagenomics, animal husbandry, MinION

## Abstract

**Introduction:**

Antimicrobial resistance (AMR) poses a significant threat to global public health. The One Health approach, which integrates human, animal, and environmental health, highlights the roles of agricultural and hospital settings in the propagation of AMR. This study aimed to analyze the resistome and gut microbiome composition of individuals from a high-intensity animal husbandry area in the western region of Santa Catarina, Southern Brazil, who were subsequently admitted to the University Hospital in the city of Florianopolis, located in the eastern part of the same state.

**Methods:**

Rectal swab samples were collected upon admission and discharge. Metagenomic sequencing and resistome analysis were employed to identify antimicrobial resistance genes (ARGs) and their associated bacterial taxa. Additionally, the impact of the hospital environment on the resistome and microbiome profiles of these patients was assessed.

**Results:**

A total of 247 genetic elements related to AMR were identified, with 66.4% of these elements present in both admission and discharge samples. Aminoglycoside resistance genes were the most prevalent, followed by resistance genes for tetracyclines and lincosamides. Notably, unique resistance genes, including *dfrF* and mutations in *gyrB*, were identified at discharge. ARGs were associated with 55 bacterial species, with *Lactobacillus fermentum*, harboring the ermB gene. (MLSB), detected in both admission and discharge samples. The most prevalent bacterial families included *Mycobacteriaceae*, Enterobacteriaceae, and *Bacteroidaceae*. Among these, *Mycobacteriaceae* was the most abundant, with ARGs primarily associated with mutations in the 16S rRNA gene, RNA polymerase subunits, and gyrases.

**Discussion:**

The study revealed a high prevalence of genes related to aminoglycoside and tetracycline resistance, with a notable increase in certain resistance determinants at discharge, likely influenced by extended antimicrobial use. The presence of *mcr* genes, associated with colistin resistance, in both admission and discharge samples from a single patient highlights a concerning trend in AMR, particularly in relation to animal husbandry. These findings underscore the substantial impact of antimicrobial use on resistance development and the complex dynamics of the resistome in hospital settings. They also emphasize the influence of local factors, such as intensive animal production, on resistance patterns and advocate for ongoing surveillance and policy development to manage multidrug-resistant bacteria eVectively.

## 1 Introduction

Antimicrobial resistance (AMR) is a natural phenomenon caused by the expression of a variety of resistance genes (Ramamurthy et al., 2022). The development of resistance is exacerbated by the high demand for antimicrobials, their misuse, and unnecessary prescriptions (Belachew et al., 2021). This situation diminishes the effectiveness of antimicrobial treatments, increasing morbidity and mortality rates of infectious diseases, leading to more complex therapeutic approaches, and escalating healthcare costs (Martínez et al., 2017). Currently, AMR is already among the top 10 threats to public health (CDC, 2019). Each year, bacterial infections are responsible for an estimated 7.7 million deaths, with 65% of these deaths being associated with antibiotic-resistant bacterial pathogens (The Lancet, 2024).

The interconnection of human, animal, and environmental health is conceptualized as One Health, an integrated approach in which AMR is relevant across its various dimensions since resistance determinants are as ubiquitous bacteria (Baquero et al., 2019). Within the human body, bacterial communities are part of a diverse ecosystem with other microorganisms and are present in all mucosal surfaces and the skin (Lawton, 2022). It is well-established that these communities (microbiotas) are fundamental for the proper functioning of nearly all human systems (MacAlpine et al., 2023; Pascale et al., 2018; Zhang et al., 2015). However, antimicrobials intended to eliminate pathogens also disrupt commensal microbiota, creating an imbalance condition known as dysbiosis. Altering the composition of a microbial community can increase its susceptibility to colonization by pathogenic bacteria, while also selecting for resistant strains. Antimicrobial resistance genes (ARGs) present in microbial communities, such as the intestinal microbiota, constitute the resistome, which exhibits inter-individual variation and correlates with several factors, including diet, travel, antimicrobial use, and the surrounding environment, such as hospitals (Crits-Christoph et al., 2022). The resistome and the human microbiota serve as a reservoir of ARGs, which can be acquired by pathogenic strains through horizontal gene transfer (HGT) (Zhang et al., 2022). Population-level variations and differences in ARG abundances in commensal microorganisms, such as those occurring during antimicrobial administration, may increase the frequency of successful transmission of these genes to pathogenic strains (Anthony et al., 2022; Crits-Christoph et al., 2022; Smillie et al., 2011).

The western region of Santa Catarina state, located in southern Brazil, is the most significant area for swine and poultry meat production in the country, generating over $2 billion in revenue annually, and is also one of the leading centers of meat production globally (Secretaria de Estado da Agricultura e Pecuária de Santa Catarina, 2023). It is well-documented that antimicrobials are extensively used in animal husbandry, and consequently, this environment is considered a major source of AMR induction (Jacobsen et al., 2023). We have previously shown that farms from this region present a high variety of ARGs in both, farms with open regimens and use of antimicrobials but also in those with closed regimens and no antimicrobial use (Beltrame et al., 2022). Moreover, we detected a colistin resistance gene, *mcr-4*, in a slurry sample from one of these farms. Unfortunately, few policies currently regulate antimicrobial use in these settings, and existing measures are mostly recommendations for reducing antimicrobial use rather than detailed plans to achieve this reduction.

In this study, we evaluated the gut microbiome and resistome composition of four hospitalized patients who are workers from the western region of Santa Catarina state, an area of intensive animal husbandry, following their admission to the University Hospital, located in the eastern region of the state.

The hospital environment is characterized by the high circulation of individuals, each associated with a unique microbiota, thereby constituting a significant reservoir of ARGs. These factors contribute to a highly complex ecosystem within healthcare facilities, which play a critical role in the ongoing AMR crisis. Thus, the rapid dissemination of resistant microorganisms and ARGs represents an escalating threat to human health, necessitating ongoing efforts to characterize and understand their transmission dynamics.

## 2 Materials and methods

### 2.1 Study setting

This study was conducted with patients admitted to the University Hospital Professor Polydoro Ernani de São Thiago (UH), affiliated with the Federal University of Santa Catarina. Established on May 2, 1980, the hospital has 208 beds for clinical and surgical treatments. On average, the hospital admits approximately 720 patients and performs 456 surgeries monthly. Additionally, the hospital employs over 2,000 staff members across various clinical, surgical, and research roles (EBSERH, 2023).

### 2.2 Study population and sample collection

**Figure 1:**
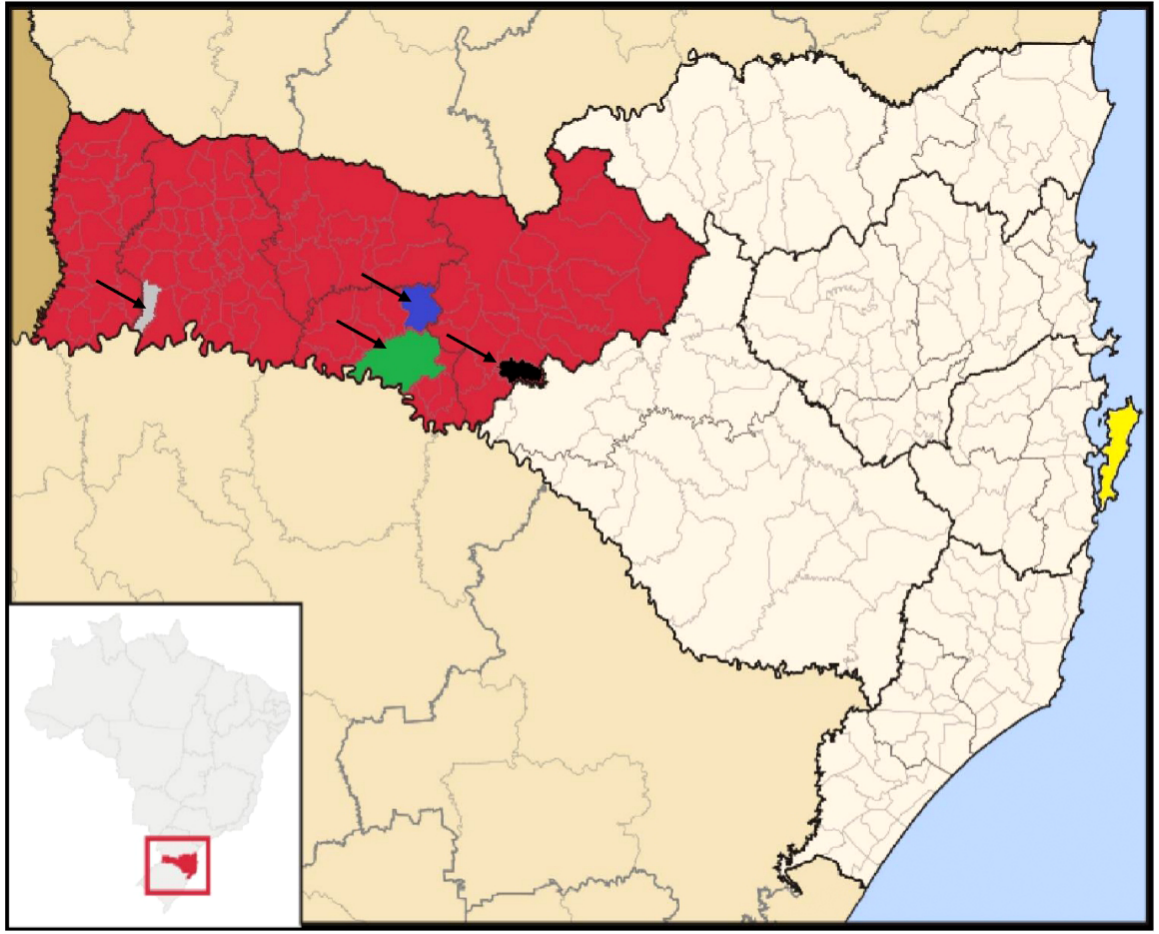
Geographical distribution of the sampled patients’ cities in the state of Santa Catarina, Brazil. The map highlights the following locations: Patient 04 - Erval Velho (black); Patient 07—Caibi (gray); Patient 09—Irani (blue); Patient 13—Concordia (green). The shaded area in red indicates the Western Santa Catarina mesoregion. The municipality of Florianopolis, where the University Hospital is located, is highlighted in yellow. Source: Adapted from "Brazil Santa Catarina location map.svg" by Raphael Lorenzeto de Abreu, available via Wikimedia Commons, licensed under CC BY 2.5 (available at https://pt.wikipedia.org/wiki/Santa_Catarina#/media/Ficheiro:Brazil_Santa_Catarina_location_map.svg).

Between August 2019 and March 2020, workers from the western region of Santa Catarina state were admitted to the UH and were recruited to participate in this study (**Figure 1**; **Supplementary Table 1**). Rectal swab samples were collected at admission (AR) and discharge (DR) from the hospital. This research was approved by the local ethics committee (Comitê de Ética em Pesquisa com Seres Humanos, CESPH-UFSC) under protocol number CEPSH/CAAE: 10282619.5.0000.0121. Flocked surface swabs (Copan, Italy) were used moistened with sterile 0.9% saline solution immediately before collection and stored in a -20 °C freezer until processing within two days.

### 2.3 Library preparation and metagenomic sequencing

DNA extraction was performed using the ZymoBIOMICSTM DNA Miniprep Kit (D6060, Zymo Research, Irving, USA), according to the manufacturer’s recommendations. The purity and quality of the DNA were verified using a NanoVueTM Plus spectrophotometer (28956058; BiochromTM, Holliston, USA).

The preparation of the metagenomic libraries was performed using the Rapid Barcoding kit (SQK-RBK004; Oxford Nanopore Technologies, Oxford, UK) following the manufacturer’s guidelines. AMPure XP magnetic beads (A63881, Beckman Coulter, Brea, California, USA) were utilized to achieve higher purity of the DNA samples. The time for each run was established in 24 h, in which we used 12 samples with different barcodes. The flow cells used were model FLO-MIN106D (R9.4.1; Oxford Nanopore Technologies) and runs were performed in a MinION sequencer (Oxford Nanopore Technologies). All sequencing runs included an internal control using the Zymo Research Community DNA Standard II (D6310).

### 2.4 Base calling and resistome construction

The base calling method performed for each race was high (processed by the Guppy offline tool, v3.4.5; Oxford Nanopore Technologies). Sequences were then submitted to the antimicrobial resistance gene identification workflow, available on the EPI2ME platform (v.2019.7.9, Oxford Nanopore Technologies; https://epi2me.nanoporetech.com/), which includes the WIMP (What’s In My Pot) tool for taxonomic identification based on Centrifuge database (Kim et al., 2016) and the ARMA pipeline (Antibiotic Resistance Mapping Application, Oxford Nanopore Technologies) which obtains gene annotation based on CARD (The Comprehensive Antimicrobial Resistance Database) (Alcock et al., 2023, 2019). Reads with qscore less than 7 were excluded.

We classified all identified genes based on the antimicrobial class that confers resistance and the mechanism of resistance. Based on the general mechanism of resistance, we classified all genes into four different categories: drug target alteration, drug efflux or drug inactivation, and others. At the class level, genes or mutations conferring resistance to more than one antimicrobial class were classified as “multiclass”. In addition, the group called “others” comprises those genes or mutations related to the classes: nitroimidazole, nucleosides, pactamycin, elfamycin, and diaminopyrimidine. All figures and statistical analyses were performed using the free online tool ResistoXplorer (Dhariwal et al., 2021).

### 2.5 Resistome and microbiome analysis

The microbiome analysis in this study was based only on species directly associated with resistance determinants, which means that only reads that were annotated to a resistance determinant were used for taxonomic identification. Taxonomic lineage data, from phylum to species, were verified according to the information provided by the NCBI Taxonomy (Schoch et al., 2020). Taxa identified as subspecies were represented only by their respective species. For reasons of graphic visualization and because they represent rare taxa, species present in only one of the analyzed samples were discarded. Microbiome composition analyses were performed based on absolute and relative frequency data of the taxa detected per sample.

## 3 Results

A total of 14 patients were recruited for our study, of which 4 were selected for further analysis. These 4 patients were specifically chosen because rectal swab samples were collected from them both at the time of admission and upon discharge. The following details pertain to these selected patients:

Patient 04 was an 85-year-old male admitted for a cutaneous infection in the left upper limb. Following a muscle drainage procedure, cultures confirmed the presence of methicillin-resistant *Staphylococcus aureus* (MRSA). The patient was treated with oxacillin and trimethoprim-sulfamethoxazole (Bactrim) during hospitalization and was subsequently discharged after a 10-day stay, following clinical improvement.

Patient 07 was a 60-year-old male admitted with an infected pressure ulcer in the sacral region. The patient received clindamycin and ceftriaxone during his hospital stay. The admission sample from Patient 07 (7AR) underwent microbiological analysis, revealing four isolates of *Escherichia coli* and one of *Providencia stuartii*. The selection of antibiotics for treatment was determined by the attending physician, independently of the data from this study. Subsequently, he was transferred to another hospital to continued care, and was discharged from the University Hospital (UH) after a 7-day admission.

Patient 09 was a 68-year-old male hospitalized for a chemotherapy cycle against blastic plasmacytoid dendritic cell neoplasm. Microbiological cultures identified the presence of *Enterococcus faecium*, *Candida* spp., and *Pseudomonas aeruginosa* resistant to cefepime and meropenem. The patient received multiple antimicrobials during his hospital stay, including trimethoprim-sulfamethoxazole, acyclovir, entecavir, cefepime, meropenem, polymyxin, voriconazole, and vancomycin. The patient died after 21 days of hospitalization.

Patient 13 was a 39-year-old male admitted for a chemotherapy cycle due to recurrent acute myeloid leukemia. He received various antimicrobials during his hospital stay, such as amoxicillin-clavulanate, sulfamethoxazole-trimethoprim, cefepime, meropenem, voriconazole, linezolid, and acyclovir. His hospitalization lasted for a total of 31 days.

### 3.1 Overall resistome analysis

The sequencing results were processed using the antimicrobial resistance gene identification workflow available on the EPI2ME platform (v.2019.7.9, ONT, https://epi2me.nanoporetech.com/). This workflow employs the ARMA (Antibiotic Resistance Mapping Application) tool to identify resistance determinants. ARMA utilizes the CARD (Comprehensive Antibiotic Resistance Database), which contains sequences of antimicrobial resistance genes and associated ontology data (Alcock et al., 2023, 2019). The results were reported considering both clinically relevant resistance determinants and those classified as not clinically relevant, according to the database.

The sequencing analysis revealed 247 different genetic elements, such as genes and punctual mutations, related to antimicrobial resistance using ARMA pipeline based on CARD database. From the 247 genetic elements pool, resistome analysis revealed that 40 (16.2%) resistance genes were exclusively found in the admission samples, while 43 (17.41%) were exclusive to the discharge samples (**Supplementary Figure 1**). The quality and yield data of the sequencing are shown in **Supplementary Table 2**. At the time of admission, the most abundantly detected exclusive genes included *H-NS*, *vatE*, a mutation in *folP*, *tet33*, and *mdtC*. At the time of hospital discharge, the most abundant exclusive genes identified were *dfrF*, a mutation in *gyrB*, *aph(6)-Id*, and *mdfA*. Additionally, 164 (66.4%) resistance genes were identified in both admission and discharge groups (**Supplementary Figure 1**).

**Figure 2:**
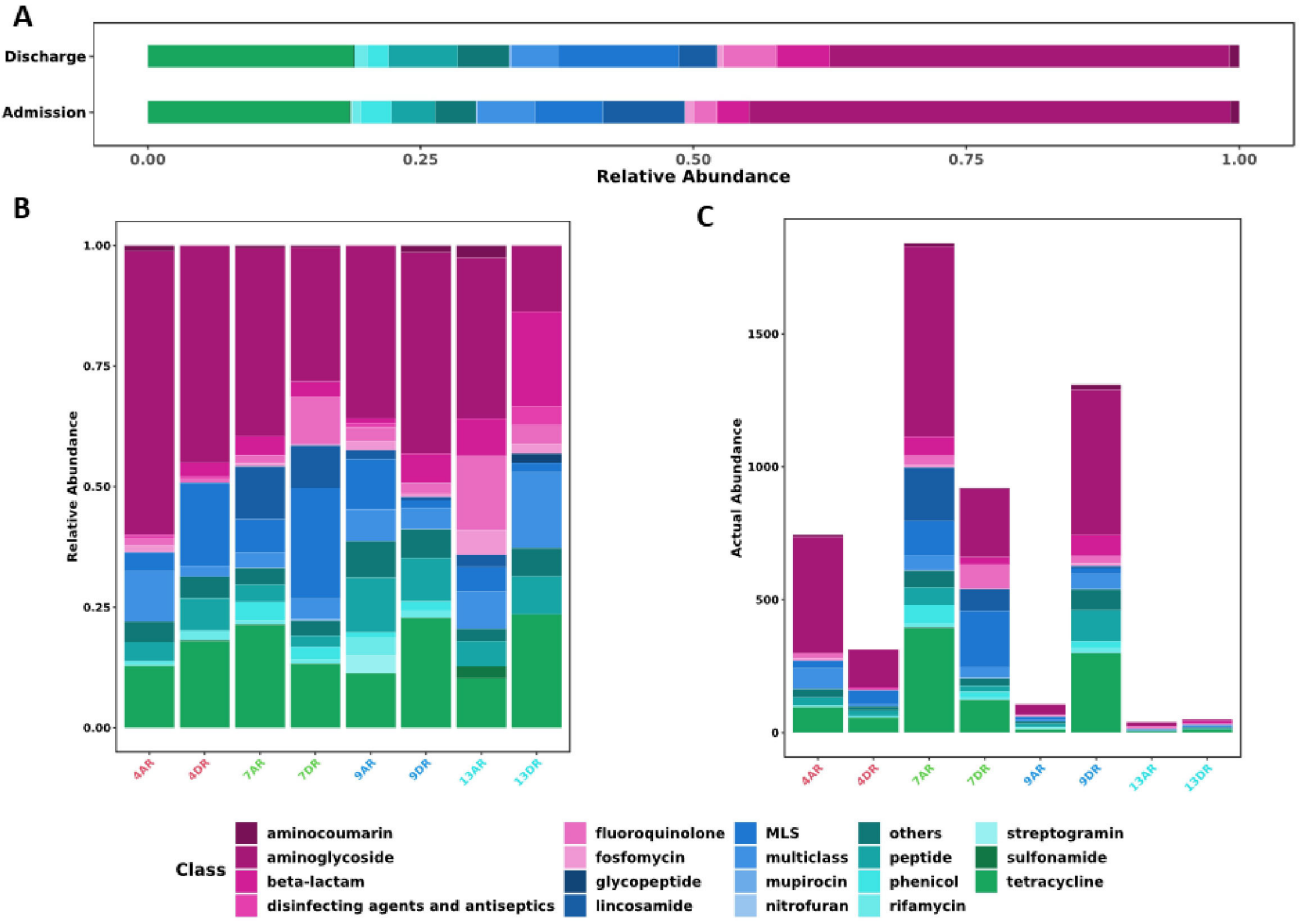
Distribution of antimicrobial resistance genes across samples by antibiotic class. **(A)** Stacked bar plot showing the relative abundance of resistance genes, grouped by antibiotic class, across sample groups. **(B)** Stacked bar plot illustrating the relative abundance of identified resistance genes in individual samples at admission (AR) and discharge (DR). **(C)** Stacked bar plot displaying the absolute number of resistance genes detected in each sample at admission (AR) and discharge (DR). Each vertical bar represents a sample, with the following color codes: Red (Patient 4), Green (Patient 7), Blue (Patient 9), and Cyan Blue (Patient 13).

The prevalence of genetic elements related to resistance against aminoglycosides was notable in both groups, primarily due to mutations in the *rrsB* gene (**Figure 2**). In the admission samples, genetic determinants conferring resistance to aminoglycosides were the most prevalent at 44.11%, followed by tetracyclines at 18.45% and lincosamides at 7.47%. Similarly, in the discharge samples, aminoglycoside resistance genes were most abundant at 36.62%, followed by tetracycline resistance genes at 18.81%, and genes exhibiting an MLS_B_ (Macrolide-Lincosamide-Streptogramin B) phenotype at 11.06% (**Figure 2**).

### 3.2 Microbiome analysis and associated antibiotic resistance genes

We conducted an analysis and classification of bacteria associated with resistance genes. The Venn diagram (**Supplementary Figure 2**) illustrates that 55 bacterial species were linked to resistance-conferring genes and mutations identified in patient samples. None of these species were unique to samples collected at the time of admission, whereas only 2 species (3.6%) were found exclusively in samples obtained at discharge: *Mycoplasma hominis* (9DR) and *Vibrio cholerae* (7DR e 9DR).

**Figure 3:**
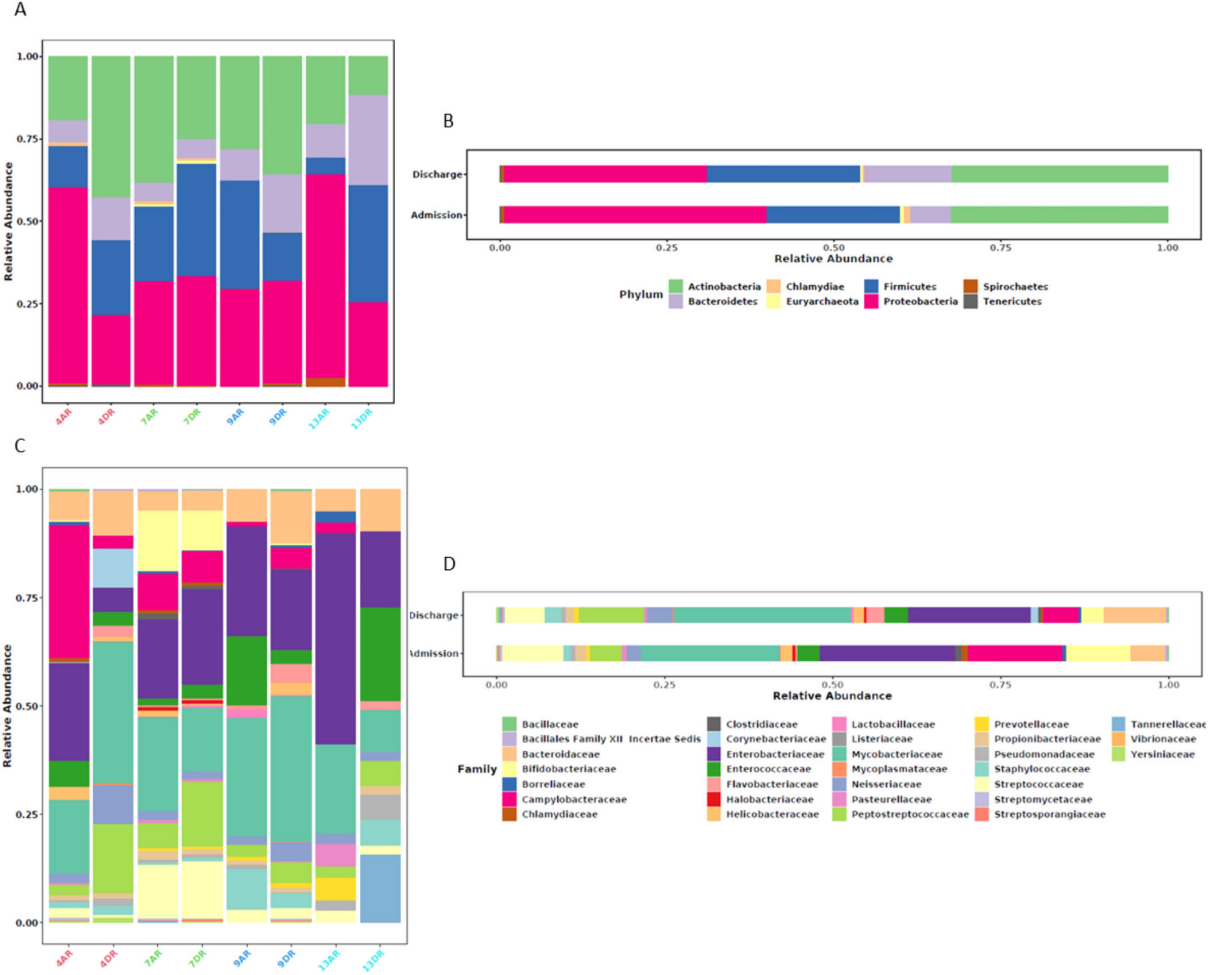
Analysis of microbiome composition by sample and by sample group, categorized by taxonomic levels of phylum **(A, B)** and family **(C, D)**. **(A, C)** show the relative abundance of microbiome profiles by individual sample, while **(B, D)** display the relative abundance by sample group. Each vertical bar represents a sample, color-coded as follows: Red for patient 4, Green for patient 7, Blue for patient 9, and Cyan Blue for patient 13.

The ten most abundant bacterial families identified in this study listed in descending order, were: *Mycobacteriaceae*, *Enterobacteriaceae*, *Campylobacteraceae*, *Streptococcaceae*, *Peptostreptococcaceae, Bacteroidaceae*, *Bifidobacteriaceae*, *Enterococcaceae*, *Neisseriaceae*, and *Staphylococcaceae*. The specific species within each family, along with the corresponding samples in which they were detected, are presented in **Figure 3**.

In the admission sample of patient 4 (4AR), the most abundant bacterial families were, in order of prevalence, *Campylobacteraceae*, *Enterobacteriaceae*, and *Mycobacteriaceae*. *Campylobacteraceae* was the most prevalent family, with the *aph(3’)-IIIa* gene being the most abundant resistance determinant, accounting for 98% of the total reads. This gene encodes a phosphotransferase that confers resistance to the aminoglycoside class of antibiotics. The second most abundant family, *Enterobacteriaceae*, predominantly featured a mutation in the 16S rRNA *rrsH* gene as the primary resistance determinant, although this mutation represented only 6% of the total reads within the family. This mutation also confers resistance to the aminoglycoside class. The third most abundant family, *Mycobacteriaceae*, contained a mutation in the 16S rRNA *rrsB* gene as the most prevalent resistance element, which also confers resistance to the aminoglycoside class.

The discharge sample from patient 4 (4DR) displayed a higher abundance of families distinct compared to the admission sample. Firstly, in this sample, the most abundant family was *Mycobacteriaceae*, with the primary resistance determinant being a mutation in the 16S rRNA gene, conferring resistance to the aminoglycoside class of antibiotics. The second most abundant family was *Peptostreptococcaceae*, represented by a single species, *Clostridioides difficile*, across all samples analyzed in this study. The only resistance determinant identified in this family, within this patient’s discharge sample, was *tetM*, which confers resistance to the tetracycline class. The third most abundant family was *Bacteroidaceae*, where the most prevalent resistance gene was *ermF*, which confers resistance to three different classes of antimicrobials, resulting in the MLS_B_ resistance phenotype.

In the admission sample from patient 07 (7AR), the three most abundant bacterial families were: *Mycobacteriaceae*, *Enterobacteriaceae*, and *Bifidobacteriaceae*. Within *Mycobacteriaceae*, the most prevalent resistance determinant was a mutation in the 16S rRNA gene, accounting for 8% of the total reads, which confers resistance to viomycin and, more broadly, to the antimicrobial peptides (AMPs) class. In *Enterobacteriaceae*, the most abundant resistance determinant was a mutation in the 16S rRNA *rrsC* gene, representing 7% of the reads and conferring resistance to the aminoglycoside class of antibiotics. Lastly, the third most abundant family, *Bifidobacteriaceae*, was characterized by the presence of a single resistance gene, *tetW*, which confers resistance to the tetracycline class of antibiotics.

In the discharge sample of patient 07 (7DR), the *Enterobacteriaceae* family remained one of the most abundant, ranking first with the resistance determinant *qnrB19*, which constituted 17% of the total reads and confers resistance to the fluoroquinolone class of antibiotics. The second most abundant family was *Peptostreptococcaceae*, with a notably high abundance of the *ermB* gene (94%), which is associated with the MLS_B_ resistance phenotype. The third most abundant family, *Mycobacteriaceae*, had its most prevalent resistance determinant as a 16S rRNA mutation, accounting for 11% of the reads and conferring resistance to the aminoglycoside class.

In the admission sample from patient 09 (9AR), *Mycobacteriaceae* was the most abundant bacterial family. This family exhibited three resistance determinants with equal read counts (14%). Two of these were mutations in the 16S rRNA *rrsB* gene, which conferred resistance to the aminoglycoside class, specifically kanamycin A and streptomycin. The third resistance determinant was also a mutation in the 16S rRNA, which conferred resistance to the antimicrobial peptides (AMPs) class. The second most abundant family in this sample was *Enterobacteriaceae*, with its most prevalent resistance determinant being a 16S rRNA mutation in the *rrsD* gene (15%), conferring resistance to the aminoglycoside class. Lastly, the third most abundant family, *Enterococcaceae*, was characterized by the presence of the *ermB* gene, which is associated with the MLS_B_ resistance phenotype.

In the discharge sample from patient 09 (9DR), *Mycobacteriaceae* remained the most abundant bacterial family. The resistance determinant with the highest number of reads in this family was a 16S rRNA mutation (14%) conferring resistance to AMPs. Similar to the admission sample, *Enterobacteriaceae* was the second most abundant family, with the previously mentioned 16S rRNA mutation being the most prevalent within the group (11%). The third most abundant family, *Bacteroidaceae*, was characterized by the *tetQ* gene, which was a significantly more abundant resistance determinant (95%) and confers resistance to the tetracycline class of antibiotics.

In the admission sample from patient 13 (13AR), *Enterobacteriaceae* was the most abundant family, presenting three resistance determinants with equal read counts (11%). These included two mutations: one in the 16S rRNA *rrnB* gene, conferring resistance to aminoglycosides, and another in the *uhpT* gene, conferring resistance to fosfomycin. Additionally, the *qnrB5* gene was identified, which confers resistance to fluoroquinolones. The second most abundant family was *Mycobacteriaceae*, with the 16S rRNA mutation being the most abundant resistance determinant, conferring resistance to aminoglycosides. *Bacteroidaceae* was the third most abundant family, with the *ermF* gene as the sole resistance determinant, responsible for the MLS_B_ resistance phenotype.

In the discharge sample from patient 13 (13DR), the *Enterococcaceae* family showed the greatest abundance. Among its resistance determinants, the most notable was the *emeA* gene (22%), which confers resistance to disinfectants and antiseptic agents. The second most abundant family was *Enterobacteriaceae*, with a 16S rRNA mutation in the *rrsB* gene being the most prevalent resistance determinant, conferring resistance to tetracyclines. *Tannerellaceae* was the third most abundant family in the sample, with the *cfxA5* gene as the most abundant resistance determinant (62%). It is noteworthy that this family is not even among the three most abundant in any other sample and was not even among the ten most abundant families in the overall study.

## 4 Discussion

The resistome composition analysis revealed a high prevalence of genes associated with resistance to aminoglycosides and tetracyclines across all samples in this study. The genetic element *aph(3’)-IIIa*, an aminoglycoside phosphotransferase encoded by plasmids in *Staphylococcus aureus* and *Enterococcus* spp., was the most prevalent among the resistance genes identified. Notably, this element accounted for 30% of the reads in the admission sample from patient 04.

The MLS_B_ phenotype, characterized by resistance to macrolides, lincosamides, and streptogramins B, is represented by the *erm* gene family, with *ermB* being the most prevalent determinant among the samples analyzed. Notably, samples from patient 07 displayed the highest abundance of this resistance determinant, with 112 reads in the admission sample (7AR) and 198 reads in the discharge sample (7DR). The resistance observed is attributed to target site modification mediated by the *erm* gene family, which encodes a methylase enzyme that methylates and modifies the antibiotic target, 23S rRNA (Manandhar et al., 2021).

The notable increase in the prevalence of the MLS_B_ phenotype observed between the admission and discharge samples of patient 07 may be attributed to the use of two antimicrobials during hospitalization: ceftriaxone, a beta-lactam antibiotic, and clindamycin, a lincosamide antibiotic.

Furthermore, we identified eleven bacterial isolates belonging to the order *Enterobacterales* in patient 07 by classical microbiological analysis, revealing four *Escherichia coli* isolates and one *Providencia stuartii* isolate upon admission. At discharge, five additional *E. coli* isolates and one *Klebsiella pneumoniae* isolate were identified. Antimicrobial susceptibility testing (AST) indicated that these isolates exhibited resistance to a range of antimicrobials. Notably, eight of the eleven isolates showed resistance to ceftriaxone, encompassing seven *E. coli* isolates and the *Providencia stuartii* isolate (data not shown). Additionally, an increase in the prevalence of fluoroquinolone resistance determinants was observed in the 7DR sample compared to 7AR, with the presence of five variants of the *qnrB* gene family.

The resistome analysis of patient 09’s samples revealed the presence of the *mcr-1* gene, which was consistently identified at both admission and discharge. In this case, we believe that it consists of an acquisition outside the hospital and a stable colonization during the hospitalization of this patient. Other longitudinal studies about *mcr-1* have already shown its prevalence over the years in patients but also in husbandry environments (Zhong et al., 2018). Notably, the *mcr-4* gene was previously detected in a swine production facility located in the same region as the patient (Beltrame et al., 2022). The *mcr* genes are significant as determinants of colistin resistance transmitted via mobile genetic elements (Gao et al., 2016; Ling et al., 2020). Colistin, a polymyxin antibiotic, is considered a last-resort treatment for infections caused by multidrug-resistant Gram-negative bacteria in humans. Although resistance to colistin was initially believed to arise solely through chromosomal point mutations, the emergence of resistance mediated by *mcr* genes on plasmids has been documented in bacteria from various sources, including animals, humans, food, farms, and the environment since 2015 (Hussein et al., 2021; Liu et al., 2016). Considering the high complexity of the horizontal transfer of genes in a microbiome context, this finding highlights the importance of new policies to avoid dissemination and further infections of bacteria that are resistant to last-resort antibiotics. It is important to consider also that bacteria tend to accumulate resistance genes through genetic recombination, making colonizing bacteria into reservoirs of genetic mobile elements of pan-resistance. This trend is concerning as it highlights the growing spread of resistance to advanced antimicrobials across different environments.

During infection, the selective pressure exerted by antimicrobials drives microorganisms to acquire mutations in genes targeted by these drugs, leading to the development of resistance. Bacterial strains possessing such mutations often exhibit enhanced proliferation compared to those lacking antimicrobial resistance genes (ARGs) (Jian et al., 2021). For instance, to patient 9 was administered sulfamethoxazole (a sulfonamide) and trimethoprim (a diaminopyrimidine) for 20 days. Notably, resistance determinants *dfrA1*, *dfrA17*, and *sul2*, which are associated with resistance to these antimicrobials, were detected exclusively in the discharge sample. This suggests that the prolonged use of these medications may have contributed to the acquisition of resistance.

Conversely, the tetracycline resistance determinant *tetQ*, which encodes a ribosomal protection protein and is associated with a conjugative transposon, was detected in seven out of the eight samples analyzed. This gene was particularly prominent in the discharge sample from patient 09 (9DR), where its abundance increased approximately 19-fold compared to the admission sample from the same patient. The association of many tetracycline resistance determinants with mobile genetic elements likely contributes to their widespread distribution across various bacterial genera, thereby explaining their significant abundance (Pavelquesi et al., 2021).

Patient 13 exhibited a greater number of resistance determinants at discharge compared to admission, although there was a decrease in aminoglycoside and beta-lactam resistance determinants. This change is attributed to the diverse antimicrobials administered during the patient’s hospitalization. Over the 31-day stay, the patient received amoxicillin-clavulanate, a penicillin-class antibiotic combined with a beta-lactamase inhibitor that broadens the antimicrobial spectrum; cefepime, a fourth-generation cephalosporin with activity against both Gram-negative and Gram-positive organisms; and meropenem, a carbapenem considered a “last-resort” treatment against infections by Gram-positive and Gram-negative bacteria, due to its resistance to extended-spectrum beta-lactamases (ESBLs) (Huttner et al., 2020; Meurant et al., 2021; Raza et al., 2021). Additionally, the patient also received trimethoprim-sulfamethoxazole and linezolid, with the former belonging to the sulfonamide class, often administered in combination with a diaminopyrimidine to inhibit enzymes in the folic acid metabolic pathway, and the latter being an oxazolidinone, effective in treating Gram-positive pathogens, including methicillin-resistant *Staphylococcus aureus* (MRSA) and vancomycin-resistant *Enterococcus* (VRE) (Roger et al., 2018; Wu et al., 2021).

Despite the small sample size, the findings of this study regarding antimicrobial resistance are consistent with existing literature. A whole-genome study of 335 novel bacterial species from the human microbiota identified MLS_B_, aminoglycosides, and tetracyclines as the most prevalent resistance determinants (Khabthani et al., 2022). Similarly, Ma et al. (2016) reported high levels of resistance genes for tetracyclines, multidrug resistance, and aminoglycosides in fecal samples from humans, chickens, and pigs. Additionally, Wang et al. (2020) discovered 330 antimicrobial resistance genes in 18 samples of human, chicken, and pig feces, conferring resistance to 21 classes of antimicrobials, with tetracyclines, MLS_B_, and aminoglycosides being among the most common.

Moreover, our results shows that aminoglycoside resistance is strongly related to bacteria from *Enterobacteriaceae, Mycobacteriaceae and Neisseriaceae* families. Other studies that look for aminoglycoside resistance genes presence in isolates from inpatients also find high prevalence in these families previously cited. A Chinese group, for example, found high prevalence of aminoglycoside-modifying enzyme genes in isolates of *Enterobacter cloacae* in a teaching hospital (Zhu et al., 2020). Another study suggests treatment alternatives to *Klebsiella pneumoniae, Pseudomonas aeruginosa* and *Acinetobacter baumannii* that are co-resistant to aminoglycosides, tetracyclines and other antimicrobials, highlighting the prevalence of these antimicrobials resistance in hospital environments (Karakonstantis et al., 2020).

Despite presenting a higher error rate than other platforms, the low capital investment required to obtain equipment such as MinION (Oxford Nanopore Technologies, Oxford, United Kingdom) contrasts with other companies available on the market, increasing its popularity. In addition, the association of this sequencing technology with refined bioinformatics pipelines highlights the potential of shotgun metagenomic sequencing with MinION (Leggett et al., 2020). Its use has proven to be efficient, user-friendly, and easy to insert into the laboratory routine. With nanopore technology, it is possible to detect antimicrobial resistance genes and their respective host microorganisms (Leggett et al., 2020).

Similar to the impact of clinical antimicrobials, the misuse of disinfectants and insufficient understanding of biosafety principles impose selective pressure, leading to the development of resistant microorganisms (Mc Carlie et al., 2020). Numerous studies have highlighted the correlation between resistance to antimicrobials and disinfectants in various bacteria, a phenomenon attributed to co-selection (Rozman et al., 2021; Staats et al., 2023; Van Dijk et al., 2022). The gene *emrD*, identified in this study, has been previously reported to encode an efflux pump that facilitates the expulsion of benzalkonium chloride from bacterial cells, thereby contributing to resistance (Nishino and Yamaguchi, 2001). Short et al. (2021) demonstrated that benzalkonium chloride, when used in high concentrations for surface disinfection in healthcare and domestic environments, interferes with the efficacy of aminoglycoside antibiotics in *Acinetobacter baumannii* and other pathogens in the ESKAPE group, such as *Escherichia coli*, *Enterobacter cloacae*, and *Klebsiella pneumoniae* (Short et al., 2021).

The microbiome composition of the analyzed samples was evaluated, revealing the predominance of the phyla *Proteobacteria*, *Actinobacteria*, and *Firmicutes*. This observation contrasts with the commonly reported dominance of *Bacteroidetes* and *Firmicutes*, followed by *Proteobacteria*. The composition and functionality of the intestinal microbiota can be influenced by various factors, including host genetics, diet, age, and antimicrobial usage (Gomaa, 2020).

Of particular note, the admission and discharge samples from patients 07 and 09 revealed the presence of *Lactobacillus fermentum* associated with the *ermB* gene, which confers the MLS_B_ resistance phenotype. Certain *Lactobacillus* species, including *L. fermentum*, are of interest due to their potential use as probiotics (Fernández et al., 2020). Clinical trials have demonstrated that daily intake of a formula containing *L. fermentum* CECT5716 by six-month-old children significantly reduced the incidence of upper respiratory tract infections, gastrointestinal infections, and overall infections in the subsequent six months (Maldonado-Lobón et al., 2015). Consequently, species within the genus *Lactobacillus*, including *L. fermentum*, have been granted Generally Recognized As Safe (GRAS) status by the FDA (Food and Drug Administration, USA) and Qualified Presumption of Safety (QPS) status by the European Food Safety Authority (EFSA) (Fernández et al., 2020).

However, literature indicates that lactobacilli exhibit inherent resistance to vancomycin, aminoglycosides, and most nucleic acid inhibitors (Gueimonde et al., 2013). Resistance to erythromycin and tetracycline is acquired, and thus the associated genetic determinants are often transferable. Acquired erythromycin resistance, encoded by the *ermB* gene, has been detected in strains of *L. fermentum* isolated from Chinese fermented foods. Additionally, plasmids potentially involved in transferring antibiotic resistance have been identified in *L. fermentum* (Anisimova and Yarullina, 2018). A study also demonstrated the successful transfer of the *ermB* gene from *L. fermentum* to *Enterococcus faecalis* (Nawaz et al., 2011). Thus, the detection of a resistance determinant in this non-pathogenic species is concerning, highlighting the need for further investigation into the antibiotic resistance profiles of probiotic cultures at both physiological and molecular levels.

In the admission samples, *Escherichia coli* and *Streptococcus agalactiae* emerged as prominent species. *E. coli*, a Gram-negative bacterium, can exist as both a commensal and pathogenic organism and is known for its broad spectrum of antimicrobial resistance, including resistance to fluoroquinolones (Fuga et al., 2022). Notably, in the United States, where fluoroquinolones are not approved for use in animal production, the resistance rates of *E. coli* to fluoroquinolones and quinolones are reported to be below 5%. In contrast, the average resistance rate in *E. coli* exceeds 40% in countries such as Brazil, China, and across the European Union, where the use of fluoroquinolones in animal production is permitted (Roth et al., 2019).

In the discharge samples, *Clostridioides difficile*, *Bacteroides fragilis*, and *Neisseria meningitidis* were notably prevalent. *N. meningitidis*, the primary causative agent of invasive meningococcal disease (IMD), including septicemia and meningitis, poses a significant health threat due to its potential for rapid progression and epidemic spread, necessitating immediate antibiotic intervention and prophylaxis (Taha and Deghmane, 2022). Although resistance in *N. meningitidis* is relatively rare, patterns of reduced susceptibility to antibiotics such as ampicillin, penicillin G, ciprofloxacin, levofloxacin, and trimethoprim-sulfamethoxazole have been documented. Furthermore, a strain of *N. meningitidis* harboring the beta-lactamase gene ROB-1 (*bla*_ROB-1_) has been recently identified in several countries (Shao et al., 2022). The identification of *N. meningitidis* associated with antimicrobial resistance genes in this study underscores the critical need for ongoing surveillance to monitor and address the emergence of resistant strains.

In our study, the most prevalent bacterial family identified was *Mycobacteriaceae*, underscoring its significance in the resistome profile observed. Within this family, resistance genes linked to aminoglycosides, peptides, rifamycins, and fluoroquinolones were detected. The presence of these resistance determinants is particularly alarming due to the critical role these antibiotic classes play in the treatment of mycobacterial infections.

**Figure 4:**
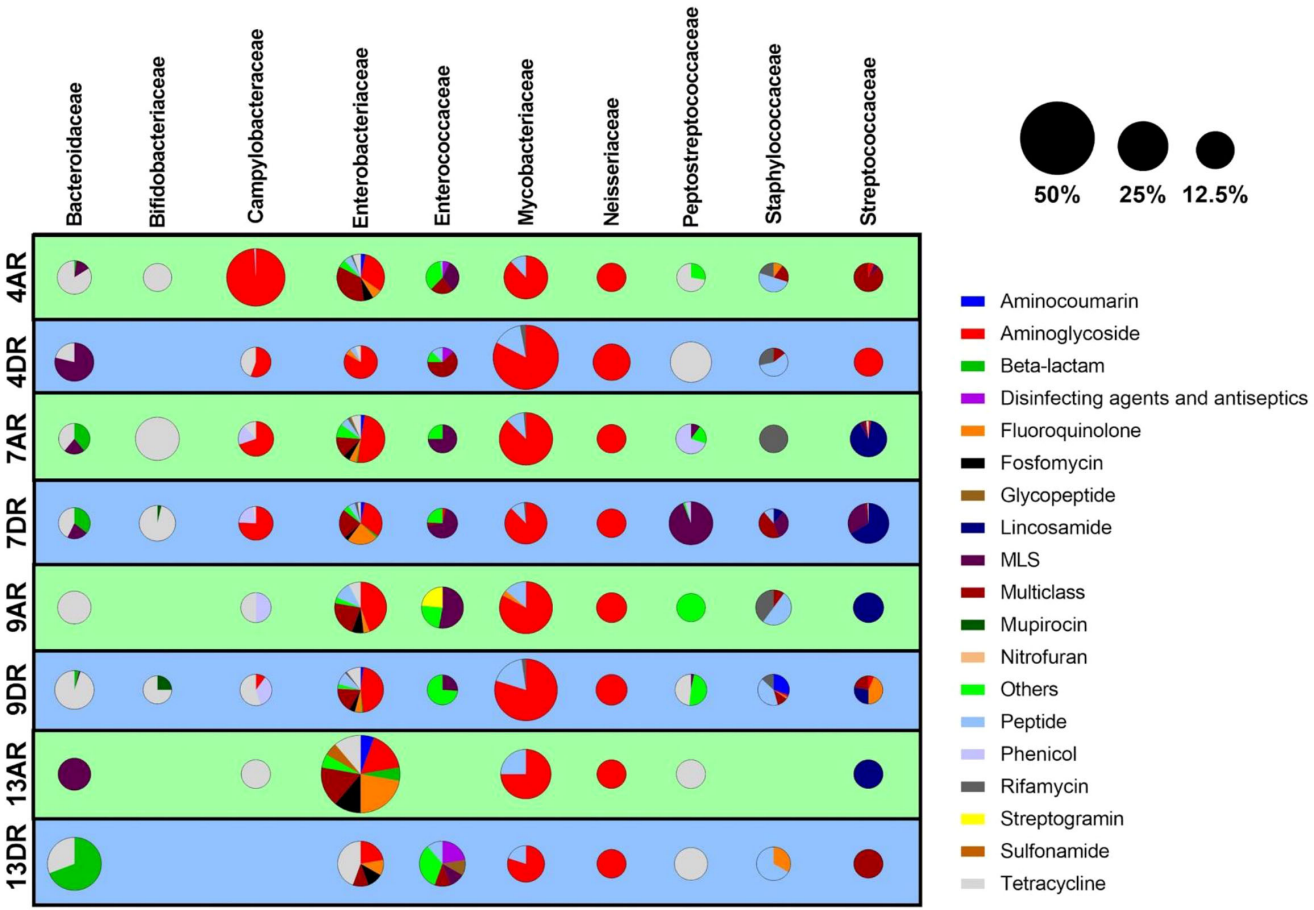
Distribution of antibiotic resistance classes across bacterial families in patient samples. Each row represents an individual sample, while each column corresponds to one of the ten most abundant bacterial families identified. For each sample-family combination, a pie chart depicts the relative abundance of various resistance classes within that bacterial family. The size of each pie chart reflects the overall abundance of the bacterial family within the microbiome of the respective sample.

Our findings reveal that the primary mechanisms of resistance are associated with mutations in genes encoding essential targets. Notably, these include mutations in 16S rRNA genes related to aminoglycoside resistance, alterations in RNA polymerase subunits associated with rifamycin resistance, and changes in DNA gyrases contributing to fluoroquinolone resistance (**Figure 4**).

In this study, we analyzed the resistome and microbiome composition of individuals admitted to and hospitalized at the University Hospital in Florianópolis, a coastal city in southern Brazil. Despite the small sample size, a key limitation of the study, the results are particularly significant due to the unique characteristics of this patient cohort. This work was part of a larger project investigating the dynamics of antimicrobial resistance gene (ARG) circulation in Santa Catarina, focusing on the interplay between human healthcare and animal husbandry environments and published in part in 2022 (Beltrame et al., 2022). All recruited patients, although hospitalized in Florianópolis, originated from the Western region of Santa Catarina, a livestock-intensive area located approximately 400–650 km inland. While we initially aimed to recruit a larger cohort, the number of eligible patients from this region was lower than anticipated, further reduced by exclusion criteria and the interruption of the study due to the COVID-19 pandemic. Moreover, our study sought to evaluate the influence of the hospital environment on the resistome and microbiome. The inclusion of admission samples allowed us to account for variations in patient age and enrich our understanding of pre-existing resistome characteristics prior to hospitalization. Firstly, the patients are permanent residents of the western region of Santa Catarina, whose capital is Florianopolis. This region is a leading global producer of swine and poultry meat, which is associated with high levels of antibiotic use in livestock. Although Brazilian legislation mandates stringent controls on antimicrobial use in animal husbandry—including the requirement for veterinary prescriptions, monitoring of antimicrobial resistance, and adherence to safety standards to safeguard public health (MAPA, 2006, 2016)—there is less focus on mitigating environmental contamination from antimicrobials used in these agricultural practices.

Secondly, residents of this region are transported by municipal vehicles for treatment and hospitalization at the University Hospital in Florianopolis, situated at the opposite end of the state. This hospital is renowned for its specialization and high quality, being affiliated with the Federal University of Santa Catarina, ranked as the fifth-best university in Brazil (UFSC, 2024). The transportation and medical treatment provided are free of charge through the Brazilian Unified Health System (SUS).

Lastly, following discharge, patients are transported back to their hometowns using municipal transport services, which, while suitable for inter-municipal travel, are not specifically designed for transporting patients or individuals in recovery.

Thus, the observations presented herein reflect a highly unique situation. This involves the transfer of individuals colonized by various types of multidrug-resistant bacteria, likely acquired in regions with intensive animal production, to a hospital of significant complexity located at the opposite end of the state of Santa Catarina. This hospital, renowned for its advanced medical care, serves numerous patients from diverse cities and municipalities within the state and beyond. These patients receive treatments involving a broad spectrum of antibiotics, potentially influencing the resistome dynamics within this specialized healthcare setting.

All these factors underscore the considerable diversity observed in both the resistome and microbiome compositions. They highlight the significant resistance selection pressures at play, which are proportional to the challenges associated with controlling the emergence of new resistances. These observations should be taken into account in the formulation of policies and the implementation of targeted measures aimed at effectively controlling the development and dissemination of novel multidrug-resistant bacteria.

## Data Availability

The data presented in the study are deposited in the BioProject repository of the National Center for Biotechnology Information, National Library of Medicine, accession number PRJNA1206727.
