# Supplementary material for: Evaluation of the resistome and gut microbiome composition of hospitalized patients in a health unit of southern Brazil coming from a high animal husbandry production region
Source: Front Antibiot. 2025 Jan 17;3:1489356. doi: 10.3389/frabi.2024.1489356 (PMC11782142; doi:10.3389/frabi.2024.1489356)
Supplement: Supplementary file 1 [file DataSheet1.pdf]

Supplementary Tables

1.2 Supplementary Tables

Supplementary Table 1. Clinical demographic data of patients admitted to the HU-UFSC-EBSERH

Patient	**Hometown**	**Admission date**	**Discharge date**	**Sample code**	**Admission unit at sampling time**
4	Erval velho	09/21/2019	10/01/2019	4AR<br>4DR	SUW<br>SUW
7	Caibi	10/18/2019	10/25/2019	7AR<br>7DR	EMG<br>EMG
9	Irani	10/22/2019	11/12/2019	9AR<br>9DR	IMW<br>IMW
13	Concórdia	01/07/2020	02/07/2020	13AR<br>13DR	IMW<br>IMW

EMG, emergency ward; IMW, internal medicine ward; SUW, surgical ward

Supplementary Table 2. Quality control, WIMP and ARMA report for all sequenced samples.

Quality Control and Barcoding						WIMP			ARMA			
Sample	qscore >7 reads (%)	Reads analyzed	Average sequence length (bp)	Total yield	Average qscore	Reads analyzed	Classified reads	Disqualified reads	Reads analyzed	Alignment	Number of identified genes	Average accuracy (%)
4AR	97,1	305934	1536	470,0 Mb	10,99	207135	140839	66296	140839	745	125	83,8
4DR	95,5	293276	1089	319,6 Mb	10,5	279717	182788	96929	182788	314	62	81,4
7AR	96,4	240514	1676	403,1 Mb	10,79	231778	48285	183493	48285	1283	173	84,4
7DR	95,3	156596	1459	228,5 Mb	10,38	149021	32592	116429	32592	919	139	87,1
9AR	99,9	729354	1600	1,2 Gb	11,31	728483	638547	89936	638547	105	53	81
9DR	99,9	233856	1926	450,6 Mb	11,17	233516	108352	125164	108352	1313	142	80,7
13AR	99,9	8454	1191	10,1 Mb	10,71	8436	3794	4642	3794	39	29	85,3
13DR	100	418692	1845	772,6 Mb	11,3	418281	356497	61784	356497	51	34	85,1

WIMP, What’s in my pot; ARMA, Antibiotic Resistance Mapping Application (Oxford Nanopore Technologies tools)
