# Supplementary material for: Evaluation of the resistome and gut microbiome composition of hospitalized patients in a health unit of southern Brazil coming from a high animal husbandry production region
Source: Front Antibiot. 2025 Jan 17;3:1489356. doi: 10.3389/frabi.2024.1489356 (PMC11782142; doi:10.3389/frabi.2024.1489356)
Supplement: Supplementary file 2 [file DataSheet2.pdf]

Supplementary Figures

1.1 Supplementary Figures

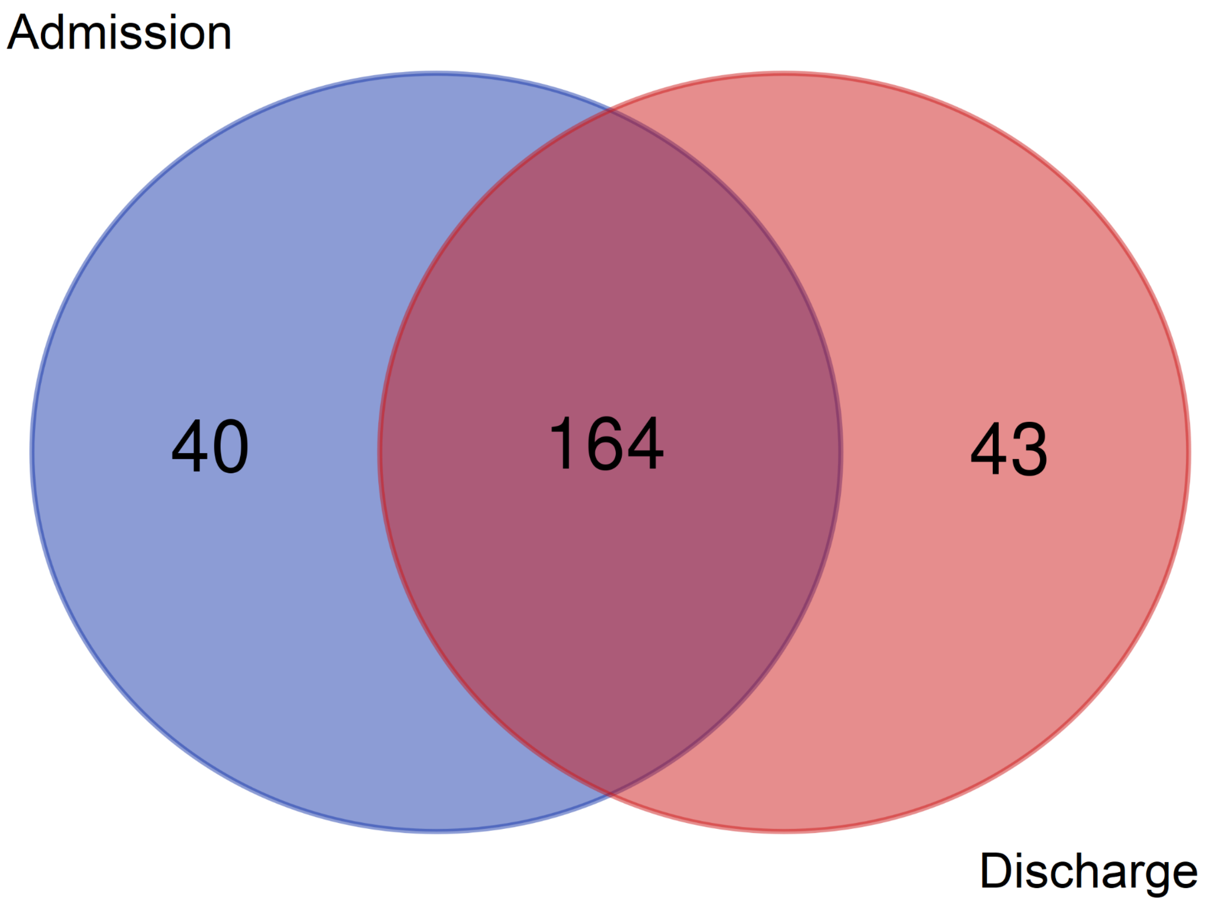

Supplementary Figure 1. Venn diagram illustrating the overlap of antimicrobial resistance genes among different sample groups. Genes are categorized into three groups: genes exclusively found in admission samples (blue), genes exclusively found in discharge samples (red), and genes common to both admission and discharge samples (purple/violet).

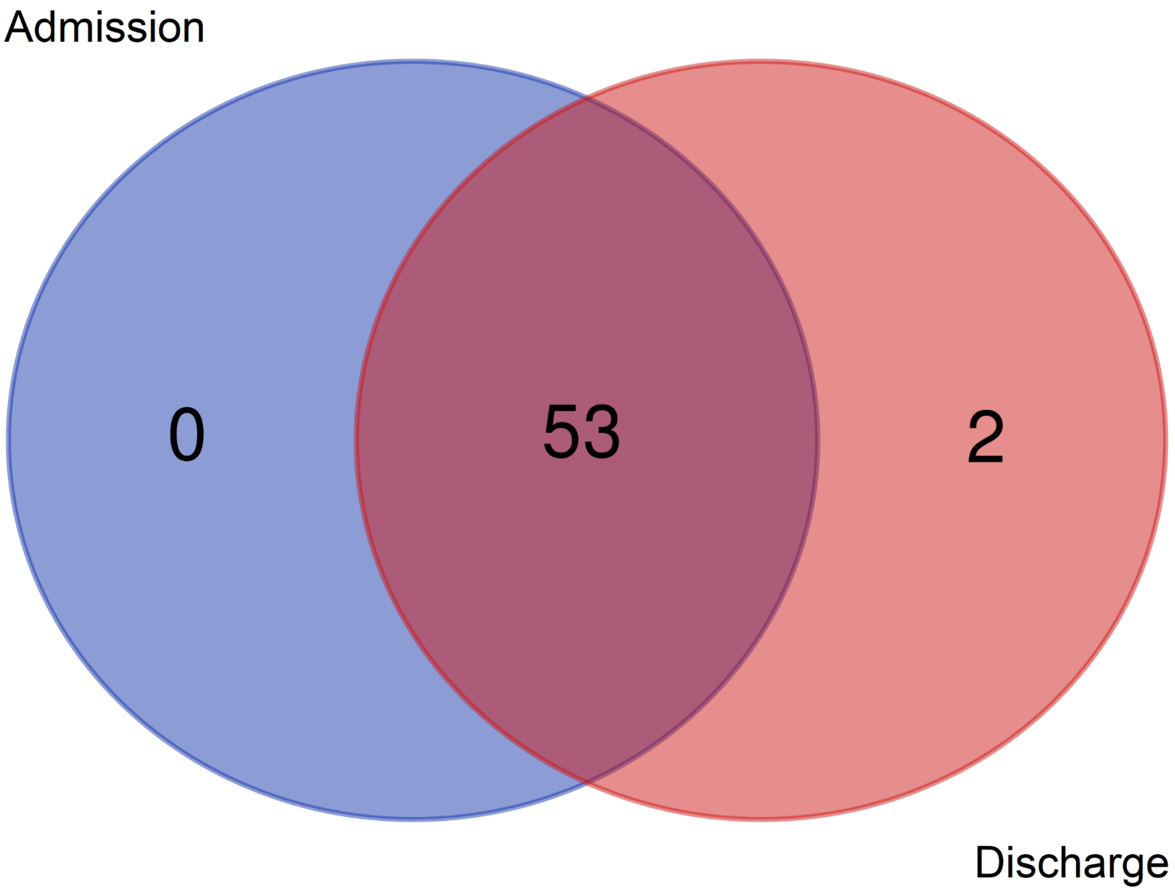

Supplementary Figure 2. Venn diagram illustrating the overlap of bacterial species in admission and discharge samples. Taxa are categorized based on their presence in the admission (blue), discharge (red) samples, or both (purple/violet).

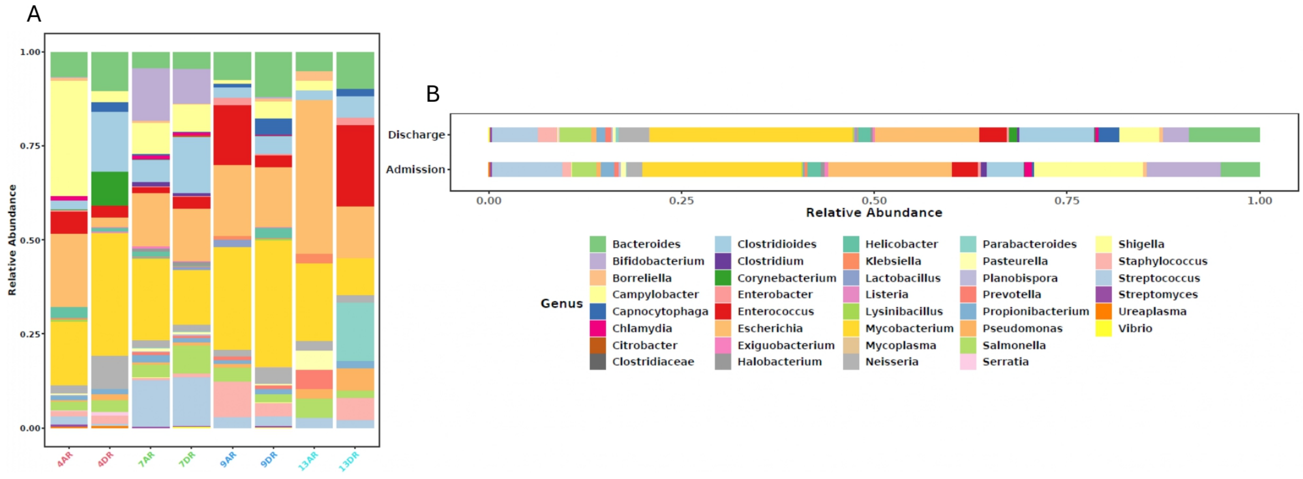

Supplementary Figure 3. Analysis of microbiome composition by sample and by sample group, categorized by taxonomic levels of genera. (A) Stacked bar plot depicting the relative abundance of 38 identified genera in individual samples collected at admission (AR) and discharge (DR). (B) display the relative abundance by sample group. Each vertical bar represents a sample, color-coded as follows: Red for patient 4, Green for patient 7, Blue for patient 9, and Cyan Blue for patient 13.

.
